# Cerebrovascular Autoregulation Monitoring in the Management of Adult Severe Traumatic Brain Injury: A Delphi Consensus of Clinicians

**DOI:** 10.1007/s12028-020-01185-x

**Published:** 2021-01-25

**Authors:** B. Depreitere, G. Citerio, M. Smith, P. David Adelson, M. J. Aries, T. P. Bleck, P. Bouzat, R. Chesnut, V. De Sloovere, M. Diringer, J. Dureanteau, A. Ercole, G. Hawryluk, C. Hawthorne, R. Helbok, S. P. Klein, J. O. Neumann, C. Robba, L. Steiner, N. Stocchetti, F. S. Taccone, A. Valadka, S. Wolf, F. A. Zeiler, G. Meyfroidt

**Affiliations:** 1 Neurosurgery, University Hospitals Leuven, Herestraat 49, B-3000 Leuven, Belgium; 2 Intensive Care Medicine, School of Medicine and Surgery, University of Milan-Bicocca, Milan, Italy; 3 Neurocritical Care Unit, National Hospital for Neurology and Neurosurgery, University College London, London, UK; 4 Barrow Neurological Institute At Phoenix Childrens Hospital, Department of Child Health/Neurosurgery, University of Arizona College of Medicine, Tucson, AZ USA; 5 Department of Neurosurgery, Mayo Clinic School of Medicine, School of Biological and Health Systems Engineering, Arizona State University, Tempe, AZ USA; 6 Department of Intensive Care, Maastricht University Medical Center, University of Maastricht, Maastricht, The Netherlands; 7 Davee Department of Neurology, Northwestern University Feinberg School of Medicine, Chicago, IL USA; 8 Grenoble Alps Trauma Center, Department of Anesthesiology and Intensive Care Medicine, Grenoble University Hospital, Grenoble, France; 9 Department of Neurological Surgery, Harborview Medical Center, University of Washington, Seattle, WA USA; 10 Anesthesiology, University Hospitals Leuven, Leuven, Belgium; 11 Department of Neurology, Barnes-Jewish Hospital, Washington University School of Medicine, St. Louis, MO USA; 12 Université Paris Sud - Hôpitaux Universitaires Paris-Sud, Paris, France; 13 Division of Anaesthesia, Department of Medicine, Addenbrooke’s Hospital, University of Cambridge, Cambridge, UK; 14 Section of Neurosurgery, University of Manitoba, Winnipeg, MB Canada; 15 Head and Neck Anaesthesia and Neurocritical Care, Institute of Neurological Sciences, Glasgow, UK; 16 Department of Neurology, Medical University of Innsbruck, Innsbruck, Austria; 17 Neurosurgery, University Hospital Brussels, Brussels, Belgium; 18 Department of Neurosurgery, University of Heidelberg, Heidelberg, Germany; 19 Policlinico San Martino, IRCCS for Oncology and Neuroscience, Genova, Italy; 20 Anesthesiology, University Hospital Basel, Basel, Switzerland; 21 Department of Clinical Research, University of Basel, Basel, Switzerland; 22 Department of Physiopathology and Transplant, Milan University and Neuro ICU Fondazione IRCCS Cà Granda Ospedale Maggiore Policlinico, Milan, Italy; 23 Department of Intensive Care, Hôpital Erasme, Université Libre de Bruxelles (ULB), Brussels, Belgium; 24 Department of Neurosurgery, Virginia Commonwealth University, Richmond, VA USA; 25 Department of Neurosurgery, University Hospital Berlin Charité, Berlin, Germany; 26 Section of Neurosurgery, Department of Surgery, Rady Faculty of Health Sciences, University of Manitoba, Winnipeg, Canada; 27 Department of Anatomy and Cell Science, Rady Faculty of Health Sciences, University of Manitoba, Winnipeg, Canada; 28 Biomedical Engineering, Faculty of Engineering, University of Manitoba, Winnipeg, Canada; 29 Centre on Aging, University of Manitoba, Winnipeg, Canada; 30 Intensive Care Medicine, University Hospitals Leuven, Leuven, Belgium

**Keywords:** Traumatic brain injury, Adult, Cerebral perfusion pressure, Cerebral blood flow, Homeostasis, Consensus development

## Abstract

**Background:**

Several methods have been proposed to measure cerebrovascular autoregulation (CA) in traumatic brain injury (TBI), but the lack of a gold standard and the absence of prospective clinical data on risks, impact on care and outcomes of implementation of CA-guided management lead to uncertainty.

**Aim:**

To formulate statements using a Delphi consensus approach employing a group of expert clinicians, that reflect current knowledge of CA, aspects that can be implemented in TBI management and CA research priorities.

**Methods:**

A group of 25 international academic experts with clinical expertise in the management of adult severe TBI patients participated in this consensus process. Seventy-seven statements and multiple-choice questions were submitted to the group in two online surveys, followed by a face-to-face meeting and a third online survey. Participants received feedback on average scores and the rationale for resubmission or rephrasing of statements. Consensus on a statement was defined as agreement of more than 75% of participants.

**Results:**

Consensus amongst participants was achieved on the importance of CA status in adult severe TBI pathophysiology, the dynamic non-binary nature of CA impairment, its association with outcome and the inadvisability of employing universal and absolute cerebral perfusion pressure targets. Consensus could not be reached on the accuracy, reliability and validation of any current CA assessment method. There was also no consensus on how to implement CA information in clinical management protocols, reflecting insufficient clinical evidence.

**Conclusion:**

The Delphi process resulted in 25 consensus statements addressing the pathophysiology of impaired CA, and its impact on cerebral perfusion pressure targets and outcome. A research agenda was proposed emphasizing the need for better validated CA assessment methods as well as the focused investigation of the application of CA-guided management in clinical care using prospective safety, feasibility and efficacy studies.

**Supplementary Information:**

The online version contains supplementary material available at. 10.1007/s12028-020-01185-x.

## Introduction

In the past decades, identifying preventable and manageable causes of secondary brain injury has led to reduced morbidity and mortality from severe traumatic brain injury (TBI). This concept of secondary insults, and the supportive evidence, have been summarized and promoted through multiple publications of guidelines and algorithms [1–4]. Low cerebral perfusion pressure (CPP) has been identified as an important secondary insult, though the literature has failed to identify clear targets of safe CPP [1, 5, 6].

Cerebrovascular autoregulation (CA) is classically defined as the maintenance of cerebral blood flow (CBF) over a wide range of CPP. Impairment of CA has long been considered a possible contributor to secondary injury [7–13]. Several methods to assess CA have been proposed and tested in clinical research, but a consensus on their accuracy, reliability and clinical utility is still lacking. These methods are based on the effect of induced alterations or spontaneous fluctuations of arterial blood pressure (ABP) on surrogate measures for CBF (e.g., intracranial pressure (ICP), the pressure–volume index; transcranial Doppler (TCD) flow velocities) or on direct CBF measurements (e.g., cortical laser Doppler flow (LDF) or perfusion imaging). The relationship of these variables with ABP or CPP is either plotted in a steady state relationship (static assessment) or by measuring the response over time (dynamic assessment) [10, 14–19]. Moving correlation coefficients between time-averaged mean TCD flow velocity and CPP (Mx) and between time-averaged mean ICP and ABP (PRx) based on waveform quality signals have been introduced, and correlations between these metrics and clinical outcomes in TBI patients were found [16, 17]. Other proposed indices correlate another variable indirectly reflecting CBF with CPP or ABP in a frequency domain that includes slow wave ABP variations. All these metrics correlate with each other and with outcome [20]. Further, computational algorithms have been developed based on PRx that enable to identify CPP zones where there is presumably intact or more efficient CA (optimal CPP or CPPopt) [21, 22].

Still, the incorporation of monitoring information on the status of CA in the management of severe TBI remains controversial. While it appears that under circumstances of raised ICP and intact CA, increasing CPP may result in reduced ICP [23], the benefit of computational calculation of dynamic CPPopt targets based on a linear mathematical interpretation of CA has yet to be demonstrated in prospective trials [24]. In addition, whether CA may be considered a physical quantity with a simply measurable metric is questionable. CBF regulation encompasses a complex and vast field of physiological processes, in which pressure-driven changes in vascular tone represent just one nonlinear element [25]. As well, regulatory mechanisms seem to be heterogeneous across the vascular tree and across brain regions [26]. At present, no single method can be regarded as the gold standard measure of CA.

The inherent validation deficit associated with clinical CA research versus the abundance of computational metrics that are appearing in literature with amazing regularity, the conceptual difference between steering CPP into its plateau region based on CPPopt algorithms versus the use of CA information for reducing raised ICP, and the absence of results from well-designed prospective clinical studies versus the referral to CA in guidelines [1, 3, 4], leave the neuro-intensive care community in a state of uncertainty. Due to all these open questions, even experts in neuro-intensive care are divided on the extent to which CA information can be implemented in clinical practice based on current knowledge.

Therefore, the aim of the current Delphi study was to develop consensus amongst expert clinicians on CA monitoring and its use in clinical practice. The eventual goal is to create clarity based on common opinions and to arrive at a common research agenda in order to facilitate progress in this promising field.

## Methods

A Delphi-method-based approach [27] was adopted to obtain consensus of the collective opinions of a group of experts. The panel was composed based on the following criteria: (1) current and active bedside clinical expertise as a physician in the acute care for adult severe TBI patients, (2) active research and/or recent publications in this field and academic appointment, (3) sufficient representation of the involved disciplines of neurosurgery, neurocritical care and neuroanesthesiology, (4) willingness, availability and ability to commit to the Delphi study on CA. The final group consisted of 25 experts, of whom 17 were neurointensivist/neuroanesthetists and 8 were neurosurgeons. In the Spring of 2019, a committee of 4 experts (GC, BD, GM, MS) carefully prepared a set of 65 statements and 12 multiple choice questions (MCQ) on CA and its potential role in the management of sedated, ventilated and ICP-monitored adults with severe TBI in the intensive care unit. The questionnaire was organized into six sections: (1) definition of CA to be used in the current Delphi consensus study (i.e., scope of the study), (2) impact of CA status on ABP and CPP management, (3) CA-based management protocols, (4) measurement of CA, (5) association of CA status with outcome and (6) recommended CA research agenda. Where applicable, participants could add comments in relation to the MCQ or statement in a free text box. The following methods to measure CA were subjected to review by the experts: Autoregulation Index (ARI) [28]; Transfer function analysis methods based on TCD flow velocity [29]; ICP response to ABP manipulation [3, 4]; PRx [17]; L-PRx [30]; LAx [31]; Mx [16]; ORx [32]; Lx [33]; TOx [34]; THx [34]; correlation of extracellular glutamate measured with microdialysis and CPP [35]; and CBFx [36]. Explanatory definitions on the methods are included in Supplementary Table 1. The full original questionnaire is available as Supplementary Text 1. The questionnaire was submitted to the experts via a web based survey (SurveyMonkey Inc., San Mateo, California, USA, www.surveymonkey.com). Experts were requested to score agreement with statements by means of a Likert scale from 1 to 8 and to answer the MCQ. Consensus was defined as >75% agreement. MCQ answers with >75% agreement and statements with >75% rating of the three lowest or three highest Likert scores were resubmitted to the experts in a second round, with the option to agree or to disagree (to confirm consensus). MCQ answers with >50% and ≤75% agreement and statements with >50% and ≤75% rating of the three lowest or three highest Likert scores were resubmitted without change along with average and personal scores (i.e., each participant could compare his individual scores with the averages of the group in order to stimulate agreement). If there was ≤50% agreement, the statement or MCQ was considered as resulting in no consensus. After two rounds, a meeting was organized at the 17th International Conference on Intracranial Pressure and Neuromonitoring in Leuven, Belgium on September 8 2019. At the meeting, all results were discussed. The group did not have the authority to change any of the results of round one and two, but did have the authority to rephrase statements when deemed appropriate, or to make a proposal regarding the relevance of emphasizing the lack of consensus on a particular statement. The responses in the comment boxes were also discussed. Rephrased statements and proposals were submitted to the expert group in a third and final web-based survey round. Statements and proposals scoring >75% agreement in round three were retained and are presented in the results section.

## Results

In round one, >75% agreement was reached in 12/77 MCQ and statements, and more than 50% and ≤75% agreement was found in 30/77 MCQ and statements. In round two, >75% agreement was reached in 19/42 MCQ and statements (consensus gain of +7), and agreement >50% and ≤75% was found in 16/42 MCQ and statements. At the face-to-face discussion in the consensus meeting, consensus was confirmed for 19 of round 2 consensus statements, and there was a decision to subtly rephrase 3 without need for new votes. More substantial rephrasing was proposed for 4 statements that had not previously reached consensus, of which one was split into two statements, and one new statement was added (see Supplementary Table 2 for rephrased statements and their rationale). In 5 instances, it was agreed to make an explicit statement that no consensus was reached. Also, 20 explanatory texts were proposed. All proposals were re-submitted to the experts in round three. Consensus was obtained for the four rephrased statements (of which one was split in two) and for the new statement, bringing the total number of consensus statements to 25 (consensus gain of + 6). There was also >75% agreement on the five proposals for making a no-consensus statement, and all explanatory texts were accepted. The full overview of 25 consensus statements, five no-consensus statements and accompanying explanatory texts in the order of the original sections can be found in Supplementary Text 2.

Table 1 summarizes all consensus statements with their scores in the different rounds, and Table 2 contains the no-consensus statements. Consensus was high on the subjects of Section 1 (scope), 2 (CPP limits), 5 (associations with outcome) and 6 (research agenda). The consensus statements in Sect. 1 reflect that the phenomenon of CA was considered from a clinical and practical perspective, rather than attempting to define its mechanisms. It is important to picture CA impairment as a potentially continuously changing process of narrowing of the CPP plateau where CBF remains stable, as well as shifting of this plateau on the CPP axis. Section 2 describes the impact of CA status on CPP management. As a result of the dynamic nature of the CBF plateau in the context of TBI and individual patient characteristics, it is impossible to define universal CPP targets that would be safe in all patients at all times. The range of the CPP target is inherently variable, but nevertheless it has boundaries that are not considered safe to violate. All experts agreed that CPP should not fall below 50 mmHg (although the real time lower boundary may lie higher). At the same time, the experts agreed that at the other extreme, high CPP is likely to be harmful too, though there are insufficient data available to define an absolute upper boundary. As for high CPP, it was argued that pharmacological measures to lower CPP (by other means than increasing sedatives) are controversial. The latest version of the Brain Trauma Foundation guidelines refers to the patient’s autoregulatory status, which may determine whether the ‘minimal optimal CPP threshold’ is closer to 60 or 70 mmHg (within the recommended 60–70 mmHg range) (1). It is important to realize that the 60–70 mmHg range simply reflects an average safe zone derived from a retrospective and static perspective at a population level, and does not take into account the dynamic nature of CA status in an individual patient. Importantly, the detrimental consequences of secondary insults relate to both intensity and duration.

**Table 1:** Statements on which consensus was reached (R1,2,3 = round 1,2,3)

No (section)	Consensus statement	Score R1	Score R2	Score R3
1 (1)	CA covers several physiological mechanisms aiming at adequate nutrient supply to the brain according to its needs. The current Delphi consensus process focuses on the clinical assessment of the ability to maintain constant global CBF in response to different external stimuli	N/A	N/A	95.8%
2 (1)	CA impairment is not binary, but a process that results in dynamic narrowing of the CBF plateau between the lower and upper limit of CA and probably also in dynamic shifts in the location of the plateau on the CPP axis	N/A	N/A	91.7%
3 (2)	A CPP below 50 mmHg should never be accepted	N/A	N/A	83.3%
4 (2)	Potential side effects of elevated CPP, such as cardiopulmonary complications and brain hyperperfusion, may occur in the higher ranges of CPP. In these ranges, additional monitoring for such side effects may be considered	N/A	N/A	100%
5 (2)	Both intensity and duration of low CPP insults are determinant in terms of association with poor outcome	83.3%	85.7%	
6 (2)	Both intensity and duration of high CPP insults are determinant in terms of association with poor outcome	83.3%	85.7%	
7 (2)	Episodes of low CPP are more detrimental than episodes of high CPP	70.8%	85.7%	
8 (2)	Because of potential dynamic CA impairment, absolute and universal CPP targets do not exist. The safe CPP zone can differ between individuals and can change within individuals	100%	100%	
9 (2)	The CPP target zone depends on CA status as well as on other variables, and is/can be narrower than the area between the lower and upper limit of CA	78.3%	100%	
10 (4)	The correlation between extracellular glutamate concentration as measured with microdialysis and CPP is inaccurate in reflecting CA	77.8%	85.7%	
11 (4)	The correlation between extracellular glutamate concentration as measured with microdialysis and CPP is not validated as a reflection of CA	76.5%	93.7%	
12 (4)	Current methods to estimate CA status are insufficiently understood. The different indices produce different information	73.9%	95.2%	
13 (4)	Information on CA status may be helpful, but is subordinate to ICP, CPP and PbO2 signals	78.3%	81.0%	
14 (5)	Impaired CA worsens tolerability for high ICP (i.e., association with worse outcome occurs at lower ICP values)	69.6%	90.5%	
15 (5)	Impaired CA worsens tolerability for low PbO2 (i.e., association with worse outcome occurs at higher PbO2 values)	59.1%	85.0%	
16 (5)	Impaired CA worsens overall tolerability for secondary insults (i.e., unfavorably shifts the thresholds associated with worse outcome)	87.0%	95.2%	
17 (5)	Whether overall CA status is intact or deficient, has an independent association with outcome (regardless of actual CPP)	78.3%	100%	
18 (6)	The priority for research on CA is high	69.6%	76.2%	
19 (6)	When a new CA assessment method is developed, it should be validated against a method that includes quantitative CBF analysis in the equation, either in animal research in the lab or in patients	N/A	N/A	91.7%
20 (6)	CA research should move to patient studies, investigating whether CA-based protocols are safe and whether they lead to different treatment strategies and different outcomes	N/A	N/A	100%
21 (6)	Research should focus on prospective patient feasibility studies to test protocols that incorporate CA information	82.6%	85.7%	
22 (6)	Research should focus on prospective patient feasibility studies to test whether dynamic CPP targets from CPPopt algorithms can be achieved/maintained	78.3%	90.5%	
23 (6)	Research should focus on prospective patient safety studies on the implementation of CA information in clinical situations	78.3%	85.7%	
24 (6)	Research should focus on prospective patient safety studies on dynamic CPP targets from CPPopt algorithms	72.7%	85.7%	
25 (6)	Research should focus on randomized controlled trials on dynamic CPP targets from CPPopt algorithms versus standard CPP management	69.6%	81.0%	

*CA* cerebrovascular autoregulation; *CBF* cerebral blood flow; *CPP* cerebral perfusion pressure; *CPPopt* optimal cerebral perfusion pressure; *ICP* intracranial pressure; *Pb02* partial pressure of oxygen in the brain tissue; *R1,2,3* round 1,2,3

**Table 2:** Statements of no consensus

No (section)	Statement of no consensus	Score expressing agreement with making a statement of no consensus (round 3)
1 (3)	There is no consensus on the manner how information on CA status should be used in clinical practice	100%
2 (4)	There is no consensus regarding sufficient accuracy of any CA assessment method that can be used in clinical practice	87.5%
3 (4)	There is no consensus regarding sufficient reproducibility of any CA assessment method used in clinical practice	87.5%
4 (4)	There is no consensus regarding sufficient validity of any CA assessment method used in clinical practice	87.5%
5 (4)	There is no consensus on the safety of implementing CA status in clinical practice	87.5%

*CA* cerebrovascular autoregulation

Section 3 deals with the implementation of information on CA status in management protocols, which is strongly related to the methods of assessing CA in Sect. 4. Little agreement existed on the accuracy, reliability and validity of the various methods that have been developed to measure CA status. The question on validity formed a central part of this discussion, especially given the absence of a gold standard to validate against. In particular, the correlation indices are based on strictly mathematical and linear interpretations of pressure reactivity. It was felt that validation of any method against patient outcome is highly relevant, but insufficient. Ideally, continuous quantitative CBF data should be in the numerator of any index. In spite of validation deficits, because PRx obtained higher scores than all other metrics in the questionnaires it was agreed that PRx to date can be considered the most accepted method to continuously monitor CA in patients and that patient research based on PRx should continue. The other correlation methods that have been proposed in the literature are not simply interchangeable with PRx. In line with the issue of CA measurement, there was also no agreement on how to safely translate CA information into clinical management protocols. This largely reflected uncertainty due to insufficient evidence. Interestingly, one of the initial statements on which no consensus could be reached was, “The implementation of estimations of CA status in clinical decisions is still a theoretical concept.” Those experts who were critical of CA monitoring approaches agreed with this statement, whereas those experts who were enthusiastic about PRx-based CPPopt algorithms disagreed.

Section 5 focuses on the association of CA status with outcome. There was a large degree of agreement on the existence of associations between CA status, tolerability for and total burden of secondary insults on outcome. In this regard, the experts also expressed a need for comprehensive integration of all different multimodality monitoring signals, without the ability to provide significant evidence-based advice at present. Finally, in Sect. 6 a research agenda was discussed. Given the important role of CA in TBI pathophysiology acknowledged by all experts, but also the validation deficit of CA assessment methods on the other hand, research priority was considered high. Even if PRx is not the perfect monitor for CA, but when protocols based on it would improve outcome, then this can be considered valuable progress. Therefore, the experts took the pragmatic decision to recommend in parallel that (1) any new methods developed should be validated against a method that includes quantitative CBF analysis in the equation; and (2) at the same time clinical CA research should move on to well-designed prospective patient studies with a focus on safety and feasibility to evaluate the computational models based on PRx.

## Discussion

The Delphi process on the clinical assessment of the ability to maintain constant global CBF in response to different external stimuli in adult ventilated, sedated and ICP-monitored severe TBI patients resulted in 25 consensus statements. Most consensus is in line with and supported by current evidence, e.g., the dynamic nature of CA impairment [21, 25, 37, 38], a lower safe threshold for CPP of 50 mmHg that should not be trespassed [39], and the importance of secondary insult duration as well as intensity [37, 40–43]. Evidence is emerging on the non-linearity and hysteresis of the CA buffer and on different physiological mechanisms explaining the lower and the upper limit of autoregulation [26, 44]. There are concerns whether this complexity can be detected by correlation indices and associated computational algorithms, although these methods seem to have potential. Several correlation methods have been proposed to date that contain different physiological information [46]. However, the central issues dominating the discussion on clinical CA assessment and its bedside use concerned the validity of the proposed methods and the availability of prospective patient data.

It has become evident that while population-based data provide a useful guideline to care, each injured individual has numerous factors that contribute to ultimate outcome. The consensus was high that understanding of the role of CA, its refinement and validation of measurement, and implementation of CA-related treatment strategies in evidence-based protocols is a promising approach, although to date it lacks enough critical literature for widespread use. Given the validation deficit of current methods to assess CA, research should invest in the development of new methods, to be validated against a method that includes quantitative CBF measurement in the equation. This should preferably start from animal models in which continuous quantitative CBF is measured under varying physiological circumstances to be then introduced at the bedside in well-designed prospective trials [44, 45]. However, redirecting focus on validation in the lab will obviously set back clinical efforts and prevent attempts to build further on the valuable parts of previous clinical work. In this regard, PRx was considered the most studied and therefore most accepted CA assessment method, in spite of lack of hard validation, concerns that it cannot capture different behavior of CA with increasing versus decreasing CPP and that interference of metabolic and oxygenation variables are insufficiently understood, poor signal to noise ratio and need for additional software not approved for medical use [26, 44, 47]. Ultimately, the lack of consensus for many of our statements was a reflection of the lack of evidence from patient studies. Therefore, there was near unanimity that the research agenda should include prospective patient feasibility and safety studies to evaluate PRx and PRx-based algorithms. We are currently eagerly awaiting the results of the Cogitate randomized trial on the automated assessment and individual implementation of a CPPopt algorithm in TBI patients that focuses on feasibility and safety, but not on resources needed. In the consensus statements, a deliberate distinction is made between the general concept of incorporation of CA information in clinical situations and the more specific application of autoregulation guided CPP (CPPopt) algorithms; both were considered equally important. This again reflects the view that research on the clinical understanding of CA and the development of new measurement and management methods should continue in parallel with patient studies using PRx as the currently most accepted CA monitoring method. The next step should then be a well-designed randomized controlled trial comparing dynamic CPP targets with standard CPP management, that—if successful—could alleviate the long-standing uncertainty as to the CPP recommendations in the adult TBI guidelines [1–4]. Before that, management decisions based on these methods should not be applied in routine care and outside of a clinical trial requiring consent. Further, additional fundamental research will remain necessary to improve our understanding of the complex, vast and fascinating field of human CBF regulation.

One limitation of the current consensus relates to the number of participants involved, which may reflect the fact that only a certain number of experts feels sufficiently comfortable to make statements on CA. Since the controversies on this topic constituted the trigger for the current project, we made sure to include all different opinions in the group. The lack of consensus on certain statements, particularly in Sects. 3 and 4, confirmed that a broad spectrum of opinions was represented in the current Delphi process, and thereby highlights its value.

The goal of the present Delphi process was to create clarity for the neuro-intensive care specialist on the use of CA in the management of adult severe TBI. The lack of validated CA assessment methods and insufficient evidence from patient studies on the incorporation in management protocols preclude any specific advice on the use of any specific method. At the same time, all experts agree on the importance of CA status in TBI pathophysiology. Although the CPP limits of the zone of constant CBF may shift, there was consensus that CPP should not drop below 50 mmHg. The monitoring of ABP and ICP is fundamental in these patients, and the monitoring of PbO2 can help to detect insults of inadequate CPP in an individual, despite CPP being above the ‘generic’ 50 mmHg threshold. Also, it is important to regularly review trends of ICP and CPP signals over several hours, which may provide a rough estimation of CA status. At most, this information may help to reduce ICP by increasing CPP in case of intact CA, not to steer CPP into the zone of active CA. In this context, the Seattle International Severe Traumatic Brain Injury Consensus Conference (SIBICC) algorithms include an ABP challenge in tier 2 for managing high ICP, in which it is proposed to titrate a vasopressor or inotrope to increase ABP by 10 mmHg for no more than 20 min [3, 4]. This simplified method of testing CA is based on a challenge study in 20 TBI patients equipped with multimodality monitoring by Rosenthal et al. [48]. It is important to emphasize that consensus was reached on the statement that information on CA status may be helpful in TBI management, but that it is subordinate to ICP, CPP and PbO2 signals. There was unanimity that, in clinical practice, a target for CPP will not only depend on CA assessment, but also on other neuromonitoring information. In practice, this means that proven and validated relationships should, for the moment, continue to have priority over CA status.

## Conclusion

Consensus amongst experts was achieved on the importance of CA status in adult severe TBI pathophysiology, the dynamic non-binary nature of CA impairment, its association with outcome and the non-existence of universal and absolute CPP targets. There was no consensus on if and how to include CA information in clinical management protocols. In spite of validation deficits and its inherent limitations, it was agreed that at present, PRx is the most accepted method to be incorporated into prospective patient safety and feasibility studies on the integration of CA information into TBI management protocols.

## Supplementary Information

Below is the link to the electronic supplementary material.

Supplementary file1 (DOCX 13 kb)

Supplementary file2 (DOCX 18 kb)

Supplementary file3 (DOCX 19 kb)

Supplementary file4 (PDF 560 kb)
