# Supplementary material for: Cerebrovascular Autoregulation Monitoring in the Management of Adult Severe Traumatic Brain Injury: A Delphi Consensus of Clinicians
Source: Neurocrit Care. 2021 Jan 25;34(3):731–8. doi: 10.1007/s12028-020-01185-x (PMC8179892; doi:10.1007/s12028-020-01185-x)
Supplement: Supplementary file 1 — Supplementary file1 (DOCX 13 kb) [file 12028_2020_1185_MOESM1_ESM.docx]

Supplementary Table 1. Explanatory definition of methods for measurement of cerebrovascular autoregulation submitted to the experts (ABP = arterial blood pressure, CBF = cerebral blood flow, CPP = cerebral perfusion pressure, Glu = glutamate, ICP = intracranial pressure, TCD = transcranial Doppler).

| Method | Explanation | Reference |
| --- | --- | --- |
| ARI | The Autoregulation Index, defined as a dimensionless index ranging from 0 to 9, representative of a response of TCD based flow velocity to a hypothetical impulse change in ABP | 28 |
| TCD transfer function analysis | Transfer function analysis methods describing the relation between ABP and TCD based flow velocity | 29 |
| ICP response to ABP manipulation | Clinical observation of ICP response to ABP manipulation as described in the SIBICC algorithms | 3,4 |
| PRx | The Pressure Reactivity Index, calculated as the moving Pearson correlation coefficient between 30 consecutive 10 second averages of ICP and mean ABP signals using waveform data capture | 17 |
| L-PRx | The 'long' Pressure Reactivity Index, calculated as the moving Pearson correlation coefficient between 20 consecutive values of ICP and mean ABP using minute by minute signal capture | 30 |
| LAx | The Low-frequencey Autoregulation Index, the averaged moving Pearson correlation coefficient of minute by minute signals of ICP and mean ABP calculated over time intervals varying between 3 and 120 minutes)[ | 31 |
| Mx | The Mean Flow Index, calculated as the Pearson correlation coefficient between 40 consecutive 6 second averages of TCD based mean flow velocity and CPP signals | 16 |
| ORx | The brain tissue oxygen pressure reactivity index, calculated as the Pearson correlation coefficient between signals of PbO2 and CPP every 30 seconds over intervals of 1, 6 or 12 hours | 32 |
| Lx | The LDF based autoregulation index, calculated as the Pearson correlation coefficient of 30 consecutive 10 second averages of LDF based CBF and CPP | 33 |
| TOx | The Total Oxygen based CA index, calculated as the moving Pearson correlation coefficient of 30 consecutive 10 second averages of Near Infrared Spectroscopy based Total Oxygen Index and CPP | 34 |
| THx | The Total Hemoglobin based CA index, calculated as the moving Pearson correlation coefficient of 30 consecutive 10 second averages of Near Infrared Spectroscopy based Total Hemoglobin Index and CPP | 34 |
| Correlation extracellular Glu with CPP | The correlation of extracellular glutamate as measured with microdialysis and CPP | 35 |
| CBFx | The thermal diffusion based autoregulation index, calculated as the Pearson correlation coefficient of 30 consecutive 10 second averages of thermal diffusion based cerebral blood flow and CPP | 36 |
