# Supplementary material for: Cerebrovascular Autoregulation Monitoring in the Management of Adult Severe Traumatic Brain Injury: A Delphi Consensus of Clinicians
Source: Neurocrit Care. 2021 Jan 25;34(3):731–8. doi: 10.1007/s12028-020-01185-x (PMC8179892; doi:10.1007/s12028-020-01185-x)
Supplement: Supplementary file 2 — Supplementary file2 (DOCX 18 kb) [file 12028_2020_1185_MOESM2_ESM.docx]

Supplementary Table 2. Overview of statements that were rephrased at the September 8 2019 consensus meeting and the rationale for rephrasing.

| No (section) | Original statement / multiple choice question | Final statement |
| --- | --- | --- |
| 1 (1) | Below you find 3 different clinical definitions of CA. Please indicate which definition you find most accurate. In case neither of the definitions conforms to your opinion, you can propose an alternative one.   - CA as defined in clinical practice refers to the components of the cerebrovascular regulatory complex, which constitute the ability to maintain stable average CBF under varying conditions of CPP and under constant PaCO2. - CA as defined in clinical practice refers to the components of the cerebrovascular regulatory complex, which constitute the ability to maintain stable average CBF under varying conditions of CPP and under varying PaCO2. - CA as defined in clinical practice refers to the complete cerebrovascular regulatory complex, which constitutes the ability to adjust and fine tune CBF. | CA covers several physiological mechanisms aiming at adequate nutrient supply to the brain according to its needs. The current Delphi consensus process focuses on the clinical assessment of the ability to maintain constant global CBF in response to different external stimuli. |
|  | Rationale: There was confusion about the use of the term ‘clinical definition’ in these statements. CA physiology covers several mechanisms and is extensive and complex, including but not limited to changes in response to activity, pressure, CO2, …. Defining or describing the physiological phenomenon of cerebrovascular autoregulation or dysregulation was not considered as a goal of this consensus exercise amongst clinical experts. Therefore, it was decided that there would be no attempt to arrive at a (clinical) definition of what CA is. However, in order to delineate the subject of the consensus effort, the following statement was put forward:  “CA covers several physiological mechanisms aiming at adequate nutrient supply to the brain according to its needs. The current Delphi consensus process focuses on the clinical assessment of the ability to maintain constant global CBF in response to different external stimuli.”  It was proposed not to change the terminology from ‘cerebrovascular autoregulation’ to ‘cerebrovascular pressure autoregulation’, as ‘pressure’ again arouses the discussion on the physiological phenomena by referring exclusively to changes in vascular tone in response to intraluminal pressure. In the online survey round 3, the above statement was presented together with a request to reply whether or not the statement was acceptable for the clinical experts. | |
| 2 (1) | N/A | New statement: CA impairment is not binary, but a process that results in dynamic narrowing of the CBF plateau between the lower and upper limit of CA and probably also in dynamic shifts in the location of the plateau on the CPP axis. |
|  | Rationale: The experts agreed that it is important to emphasize to the non-expert community that CA impairment does not work as an on/off phenomenon and that it has a dynamic nature, meaning that both the width of the plateau can be altered as well as the location of the plateau on the CPP axis. In the online survey round 3, the statement was presented together with a request to reply whether or not the expert agreed with it. | |
| 3 (2) | CPP should be kept **above**... at all times:   - 40 mmHg - 50 mmHg - 60 mmHg - 70 mmHg - other | A CPP below 50 mmHg should never be accepted. |
|  | Rationale: All experts in round 2 agreed that CPP should not go below 50 mmHg. The statement was therefore reformulated into “A CPP below 50 mmHg should never be accepted”. In the online survey round 3, the statement was presented together with a request to reply whether or not the expert agreed with it. The explanatory notes of the statement clarify that the statement is valid under the assumption that the arterial blood pressure transducer is at the height of the external auditory meatus, as well as that the statement is not in conflict with statement 8, but that it defines the absolute lower border of the zone in which the safest CPP (i.e. target CPP) varies. | |
| 4 (2) | CPP should be kept **below**... at all times:   - 100 mmHg - 90 mmHg - 80 mmHg - 70 mmHg - other | Potential side effects of elevated CPP, such as cardiopulmonary complications and brain hyperperfusion, may occur in the higher ranges of CPP. In these ranges, additional monitoring for such side effects may be considered. |
|  | Rationale: There was no consensus on the value of the absolute upper border of the zone in which the safest CPP (i.e. target CPP) varies. There was general consensus that pharmacological measures to lower CPP (by other means than increasing sedatives) are controversial. However, the experts do think that higher ranges of CPP may also be harmful to the brain. It was decided to reformulate the statement in “Potential side effects of elevated CPP, such as cardiopulmonary complications and brain hyperperfusion, may occur in the higher ranges of CPP. In these ranges, additional monitoring for such side effects may be considered.” In the online survey round 3, the above statement was presented together with a request to reply whether or not the expert agreed with it. The explanatory notes of this statement clarify that this statement is in line with statement 3 (lower border), but that there are insufficient data available to define an absolute upper border of the zone in which the safest CPP (i.e. target CPP) varies. | |
| 5 (2) | For episodes of low CPP, the intensity (depth) **AND** duration are equally determinant in terms of association with poor outcome. | Both intensity and duration of low CPP insults are determinant in terms of association with poor outcome. |
|  | Rationale: There was some discussion on the initial phrasing ‘equally’ and what it is supposed to mean. It does not mean that depth and duration are exactly and precisely equally determinant in terms of association with poor outcome, but that there is no evidence to support that either depth or duration is clearly more determinant. It was decided to rephrase ‘equally’ to ‘both’. There was no question on this statement in online survey round 3, as consensus already existed. | |
| 6 (2) | For episodes of high CPP, the intensity (level of elevation) **AND** duration are equally determinant in terms of association with poor outcome. | Both intensity and duration of high CPP insults are determinant in terms of association with poor outcome. |
|  | Rationale: There was some discussion on the initial phrasing ‘equally’ and what it is supposed to mean. It does not mean that depth and duration are exactly and precisely equally determinant in terms of association with poor outcome, but that there is no evidence to support that either depth or duration is clearly more determinant. It was decided to rephrase ‘equally’ to ‘both’. Also, the relation of statement 6 with statement 4 was discussed. There is no conflict between the acceptance of statement 6 in that insults of elevated CPP exist in terms of both level of elevation and duration on the one hand and there being no consensus on the absolute upper border of the playground in which safe CPP can vary. There was no question on this statement in online survey round 3, as consensus already existed. | |
| 12 (4) | Current tools to estimate CA status are insufficiently understood. The different indices produce different information. | Current methods to estimate CA status are insufficiently understood. The different indices produce different information. |
|  | Rationale: In the discussion, it was commented that we should replace the word ‘tool’ by ‘method’ and specify what we mean by ‘methods’. The term ‘method’ refers to the combination of at least two measured physiological values or signals, one being a surrogate for CBF and the other reflecting arterial blood pressure and its fluctuations, and the software analyzing the relation between both (either in the time or in the frequency domain). There was no question on this statement in online survey round 3, as consensus already existed. | |
| 19 & 20 (6) | For a clinical measurement/monitoring method to represent CA requires:   - validation against pial arteriolar diameter and flow velocity changes in an animal model. - validation against mathematical models of CA. - validation against outcome in prospective patient cohorts. | This was split into two statements:   - When a new CA assessment method is developed, it should be validated against a method that includes quantitative CBF analysis in the equation, either in animal research in the lab or in patients. - CA research should move to patient studies, investigating whether CA-based protocols are safe and whether they lead to different treatment strategies and different outcomes. |
|  | Rationale: This question is on the validation of CA assessment methods. As there is no gold standard, the question is against what methods should be compared to validate them as sufficiently representative of CA. The answers to the question fell apart in two categories (validation against patient outcome and validation against methods including quantitative CBF) and reflected different interpretations of the question rather than different views. Also, there was a fear that admitting validation gaps would substantially delay or even stop clinical research, while PRx is available as a promising method. In section 4, PRx was considered insufficiently validated, but still the best studied and most accepted method. It was also mentioned that, suppose that PRx does not reflect CA at all, if PRx changes management in a way that it improves outcome, this is considered valuable progress. At the end of the discussion the experts agreed that we should proceed with clinical research in the form of patient safety studies and feasibility studies based on PRx as best available method at present. At the same time, the experts agreed that any new emerging method that claims to assess CA, should be validated against a method that includes quantitative CBF analysis in the equation, either in animal research in the lab or in patients. It was agreed that comparison against/correlation with PRx is not sufficient as a validation of newly developed methods (i.e. PRx is not a gold standard). It was proposed to reformulate this question into two new statements, and to request from the experts that they indicated whether or not they agreed with the statements in online round 3. | |
