# Supplementary material for: Cerebrovascular Autoregulation Monitoring in the Management of Adult Severe Traumatic Brain Injury: A Delphi Consensus of Clinicians
Source: Neurocrit Care. 2021 Jan 25;34(3):731–8. doi: 10.1007/s12028-020-01185-x (PMC8179892; doi:10.1007/s12028-020-01185-x)
Supplement: Supplementary file 3 — Supplementary file3 (DOCX 19 kb) [file 12028_2020_1185_MOESM3_ESM.docx]

**Supplementary text 1: Overview of consensus statements and statements on lack of consensus**

Below, all consensus statements are expressed, accompanied by their scores in the subsequent rounds and with explanatory text (when decided). The statements are represented in the same order in which they were subjected to the experts, respecting the same sections. Statements on lack of consensus are similarly included in the sections in their original order.

***Section 1: definition of CA for clinical use***

*Consensus statement 1 (R1: N/A, R2: N/A, R3: 95.8%)*

CA covers several physiological mechanisms aiming at adequate nutrient supply to the brain according to its needs. The current Delphi consensus process focuses on the clinical assessment of the ability to maintain constant global CBF in response to different external stimuli.

Although the formulation of a definition or description of CA was not considered to be the goal of this Delphi project, the experts delineated its subject by the above statement.

*Consensus statement 2 (R1: N/A, R2: N/A, R3: 91.7%)*

CA impairment is not binary, but a process that results in dynamic narrowing of the CBF plateau between the lower and upper limit of CA and probably also in dynamic shifts in the location of the plateau on the CPP axis.

***Section 2: impact of CA status on ABP and CPP management***

*Consensus statement 3 (R1: N/A, R2: N/A, R3: 83.3%)*

A CPP below 50 mmHg should never be accepted.

The statement is valid under the assumption that the arterial blood pressure transducer is at the height of the external auditory meatus. Statement 2 is not in conflict with statement 8, but it defines the absolute lower border of the zone in which the safest CPP (i.e. target CPP) varies.

*Consensus statement 4 (R1: N/A, R2: N/A, R3: 100%)*

Potential side effects of elevated CPP, such as cardiopulmonary complications and brain hyperperfusion, may occur in the higher ranges of CPP. In these ranges, additional monitoring for such side effects may be considered.

The statement is in line with statement 2 on the lower border, but there are insufficient data available to define an absolute upper border of the zone in which the safest CPP (i.e. target CPP) varies.

*Consensus statement 5 (R1: 83.3%, R2: 85.7%)*

Both intensity and duration of low CPP insults are determinant in terms of association with poor outcome.

The statement does not mean that depth and duration are exactly and precisely equally determinant in terms of association with poor outcome. There is no evidence to support that either depth or duration is clearly more determinant.

*Consensus statement 6 (R1: 83.3%, R2: 85.7%)*

Both intensity and duration of high CPP insults are determinant in terms of association with poor outcome.

The statement does not mean that level of elevation and duration are exactly and precisely equally determinant in terms of association with poor outcome. There is no evidence to support that either level of elevation or duration would be clearly more determinant. Also, there are thoughts amongst the experts that elevated CPP can be harmful to the brain and that both duration and level of elevation matter in this regard, but there are no data (and hence no consensus) on an absolute upper border of the zone in which the safest CPP varies.

*Consensus statement 7 (R1: 70.8%, R2: 85.7%)*

Episodes of low CPP are more detrimental than episodes of high CPP.

The statement refers to similar magnitudes of deviation from safe CPP and is based on the given that there is insufficient data that allows to define an absolute upper border to the zone in which safest CPP varies. Still, the experts do emphasize that high CPP in itself can be harmful.

*Consensus statement 8 (R1:100%, R2: 100%)*

Because of potential dynamic CA impairment, absolute and universal CPP targets do not exist. The safe CPP zone can differ between individuals and can change within individuals.

*Consensus statement 9 (R1: 78.3%, R2: 100%)*

The CPP target zone depends on CA status as well as on other variables, and is/can be narrower than the area between the lower and upper limit of CA.

The experts understand and express by this statement that in clinical practice, a target for CPP will not only depend on CA assessment.

***Section 3: CA based management protocols***

Statement of *no consensus 1 (R1: N/A, R2: N/A, R3: 100%)*

There is no consensus on the manner how information on CA status should be used in clinical practice.

The experts do agree that CA status matters in the pathophysiology of severe TBI, but the lack of sufficient evidence on how CA status information can be used prevents the experts from issuing consensus advice. The issue is considered important enough to have a place in the research agenda.

***Section 4: measurement of CA***

*Statement of no consensus 2 (R1: N/A, R2: N/A, R3: 87.5%)*

There is no consensus regarding sufficient accuracy of any CA assessment method that can be used in clinical practice.

PRx – regardless of the absence of consensus – stood out as the method that reached most agreement and therefore, may be the best available and ‘most accepted’ method so far.

*Statement of no consensus 3 (R1: N/A, R2: N/A, R3: 87.5%)*

There is no consensus regarding sufficient reproducibility of any CA assessment method used in clinical practice.

PRx stood out as the method that reached most agreement and therefore, may be the best available and ‘most accepted’ method so far. As it is the most reproducible method available, the experts consider it the best method to base further clinical research on.

*Statement of no consensus 4 (R1: N/A, R2: N/A, R3: 87.5%)*

There is no consensus regarding sufficient validity of any CA assessment method used in clinical practice.

Nevertheless, PRx stood out as the method that reached most agreement and therefore, can be considered the best available and ‘most accepted’ method to proceed with in further clinical studies.

*Consensus statement 10 (R1: 77.8%, R2: 85.7%)*

The correlation between extracellular glutamate concentration as measured with microdialysis and CPP is inaccurate in reflecting CA.

This statement does not imply any negative judgment on the value of extracellular glutamate concentration measurement through microdialysis as a metabolic variable in the monitoring of severe TBI.

*Consensus statement 11 (R1: 76.5%, R2: 93.7%)*

The correlation between extracellular glutamate concentration as measured with microdialysis and CPP is not validated as a reflection of CA.

This statement does not imply any negative judgment on the value of extracellular glutamate concentration measurement through microdialysis as a metabolic variable in the monitoring of severe TBI.

*Consensus statement 12 (R1: 73.9%, R2: 95.2%)*

Current methods to estimate CA status are insufficiently understood. The different indices produce different information.

The term ‘method’ refers to the combination of at least two measured physiological values or signals, one being a surrogate for CBF and the other reflecting ABP and its fluctuations, and the software analyzing the relation between both (either in the time or in the frequency domain). The first sentence expresses that the methods are insufficiently validated to reflect actual CA, but emphasis is on the second sentence, i.e. the different methods are not mutually interchangeable as they are based on different physiological signals used as a surrogate for CBF. This does not mean that the information therein is not potentially valuable.

*Statement of no consensus 5 (R1: N/A, R2: N/A, R3: 87.5%)*

There is no consensus on the safety of implementing CA status in clinical practice.

In the research agenda, the experts urge for safety studies. The experts acknowledge that PRx is the ‘best available’ assessment tool and that the PRx-based CPPopt algorithm is the best advanced method to subject to patient safety studies.

*Consensus statement 13 (R1: 78.3%, R2: 81.0%)*

Information on CA status may be helpful, but is subordinate to ICP, CPP and PbO2 signals.

***Section 5: association of CA status with outcome***

*Consensus statement 14 (R1: 69.6%, R2: 90.5%)*

Impaired CA worsens tolerability for high ICP (i.e. association with worse outcome occurs at lower ICP values.

*Consensus statement 15 (R1: 59.1%, R2: 85.0%)*

Impaired CA worsens tolerability for low PbO2 (i.e. association with worse outcome occurs at higher PbO2 values).

*Consensus statement 16 (R1: 87.0%, R2: 95.2%)*

Impaired CA worsens overall tolerability for secondary insults (i.e. unfavorably shifts the thresholds associated with worse outcome).

*Consensus statement 17 (R1: 78.3%, R2: 100%)*

Whether overall CA status is intact or deficient, has an independent association with outcome (regardless of actual CPP).

***Section 6: CA research agenda***

*Consensus statement 18 (R1: 69.6%, R2: 76.2%)*

The priority for research on CA is high.

The need to move to clinical trials is highlighted.

*Consensus statement 19 (R1: N/A, R2: N/A, R3: 91.7%)*

When a new CA assessment method is developed, it should be validated against a method that includes quantitative CBF analysis in the equation, either in animal research in the lab or in patients.

*Consensus statement 20 (R1: N/A, R2: N/A, R3: 100%)*

CA research should move to patient studies, investigating whether CA-based protocols are safe and whether they lead to different treatment strategies and different outcomes.

The experts agree that we should proceed with clinical research in the form of patient safety studies and feasibility studies based on PRx as best available method at present (see also statement of no consensus 2,3,4,5). At the same time, the experts agree that any new emerging method that claims to assess CA, should be validated against a method that includes quantitative CBF analysis in the equation, either in animal research in the lab or in patients. It is agreed that comparison against/correlation with PRx is not sufficient as a validation of newly developed methods (i.e. PRx is not a golden standard).

*Consensus statement 21-25*

Research should focus on:

- prospective patient feasibility studies to test protocols that incorporate CA information *(R1: 82.6%, R2: 85.7%)*
- prospective patient feasibility studies to test whether dynamic CPP targets from CPPopt algorithms can be achieved/maintained *(R1: 78.3%, R2: 90.5%)*
- prospective patient safety studies on the implementation of CA information in clinical situations *(R1: 78.3%, R2: 85.7%)*
- prospective patient safety studies on dynamic CPP targets from CPPopt algorithms *(R1: 72.7%, R2: 85.7%)*
- randomized controlled trials on dynamic CPP targets from CPPopt algorithms versus standard CPP management *(R1: 69.6%, R2: 81.0%)*

There is a distinction between the more general notion of incorporation of CA information in clinical situations on the one hand and the concrete algorithm of CPPopt to do this on the other hand. This distinction reflects the parallel tracks view:

- the experts agree that CA status matters, but that we lack precise and validated methods to assess it and implement it; this should prompt new methods to turn to the basics and validate against methods that include quantitative CBF;
- the experts take a pragmatic view in their judgment that PRx is not perfect but it is the best available method and it should be used to move to patient studies.
