# Supplementary material for: Cerebrovascular Autoregulation Monitoring in the Management of Adult Severe Traumatic Brain Injury: A Delphi Consensus of Clinicians
Source: Neurocrit Care. 2021 Jan 25;34(3):731–8. doi: 10.1007/s12028-020-01185-x (PMC8179892; doi:10.1007/s12028-020-01185-x)
Supplement: Supplementary file 4 — Supplementary file4 (PDF 560 kb) [file 12028_2020_1185_MOESM4_ESM.pdf]

Delphi consensus round 1 on the use of cerebrovascular autoregulation monitoring in adult severe TBI management

Introduction: CPP and autoregulation in patients with severe TBI

Over the past decades, management of severe traumatic brain injury (TBI) has mainly focused on the early detection, prevention and management of secondary insults (see Brain Trauma Foundation (BTF) guidelines).

Low cerebral perfusion pressure (CPP) has been identified as a secondary insult, but literature has failed to identify a single unambiguous CPP threshold. Cerebrovascular autoregulation (CA; also referred to as cerebral autoregulation or pressure autoregulation) has been defined as the physiological mechanism that preserves cerebral blood flow (CBF) over a wide range of CPPs, through homeostatic change in cerebral vascular resistance. Thus, assuming that CPP changes provide the stimulus for CA, no or limited change in flow would be anticipated as long as the CPP remained within the upper and lower limits of autoregulation (resp. ULA and LLA).

Observational trials have demonstrated that CA is often impaired after severe TBI. The LLA and ULA may shift towards higher/lower values. In extreme cases, the CA plateau may even be absent. In addition, the degree of CA impairment may vary within the same patient, meaning that the LLA and ULA change with time (1).

The observed dynamic impairment of CA could be a plausible explanation for the failure to identify a fixed and universal CPP threshold associated with poor outcome. Moreover, the association of CPP and outcome may be better conceptualized by an intensity-duration concept, rather than by the crossing of a certain threshold alone (2). These concepts are likely to be valid for too low as well as too high CPP.

The incorporation of monitoring the CA status in the management of severe TBI is a hot topic, and is mentioned in the BTF guidelines.

BTF 2016 extract:
"Level IIB: The recommended target CPP value for survival and favorable outcomes is between 60 and 70 mmHg. Whether 60 or 70 mmHg is the minimum optimal CPP threshold is unclear and may depend on the patient's autoregulatory status."

It is plausible that monitoring the status of CA, or determining the CPP zone in which CA is preserved, could help in determining safe CPP zones (3,4). However, the current evidence is mainly retrospective, and prospective trials are lacking to support more specific guidelines on how CA could be monitored or how TBI management could be optimized when the CA status is known.

In the absence of such evidence, a RAND-based Delphi consensus methodology will be used, and questionnaires will be sent to a panel of experts, with the aim to identify valid statements representing current knowledge, as well as to guide the direction of future research.

In the next pages, you will find a set of statements on static as well as dynamic assessment of CA for the management of severe TBI. All statements relate to the setting of adult patients with severe TBI. Please either choose a statement in case of a multiple choice question or give the statement a score when requested. In the latter case, an 8-point Likert scale is used (1 Very strongly disagree; 2 Strongly disagree; 3 Disagree; 4 Slightly disagree; 5 Slightly agree; 6 Agree; 7 Strongly agree; 8

Very strongly agree). Statements with >75% agreement (MCQ) and statements with >75% rating of the three lowest or the three highest scores shall be withheld in the second round. The output of round 2 shall subsequently be discussed in a consensus meeting at ICP2019 in Leuven on September 8 2019.

1. ***Sviri GE et al. J Neurosurg 2009; 111:695-700***
2. ***Güiza et al. J Neurotrauma 2017; 34:2425-2431***
3. ***Aries et al. Crit Care Med 2012; 40:2456-2463***
4. ***Depreitere et al. J Neurosurg 2014; 120:1451-1457***

Delphi consensus round 1 on the use of cerebrovascular autoregulation monitoring in adult severe TBI management

Clinical definition of cerebrovascular autoregulation (CA)

* 1. Below you find 3 different clinical definitions of CA. Please indicate which definition you find most accurate. In case neither of the definitions conforms to your opinion, you can propose an alternative one.

- ◯ CA as defined in clinical practice refers to the components of the cerebrovascular regulatory complex, which constitute the ability to maintain stable average CBF under varying conditions of CPP and under constant $PaCO_2$.
- ◯ CA as defined in clinical practice refers to the components of the cerebrovascular regulatory complex, which constitute the ability to maintain stable average CBF under varying conditions of CPP and under varying $PaCO_2$.
- ◯ CA as defined in clinical practice refers to the complete cerebrovascular regulatory complex, which constitutes the ability to adjust and fine tune CBF.
- ◯ N/A (not within my expertise)
- ◯ Alternative (please specify)

* 2. Please rate the definition you have chosen/proposed.

Very bad							Very good	N/A (not within my expertise)
◯	◯	◯	◯	◯	◯	◯	◯	◯

Delphi consensus round 1 on the use of cerebrovascular autoregulation monitoring in adult severe TBI management

ABP and CPP management and role of impaired/intact cerebrovascular autoregulation (CA)

Please note that all questions relate to the setting of <u>adult severe TBI.</u>

* 3. CPP should be kept **above** ... at all times.

- 40 mmHg
- 50 mmHg
- 60 mmHg
- 70 mmHg
- Other (please specify)

* 4. CPP should be kept **below** ... at all times.

- 100 mmHg
- 90 mmHg
- 80 mmHg
- 70 mmHg
- Other (please specify)

* 5. An episode of low CPP has a certain intensity (depth), and a certain duration. Please indicate which of both you rate as most determinant in terms of association with poor outcome.

- For episodes of low CPP, the **intensity** (depth) is more determinant than the duration in terms of association with poor outcome.
- For episodes of low CPP, the **duration** is more determinant than the intensity (depth) in terms of association with poor outcome.
- For episodes of low CPP, the intensity (depth) **AND** duration are equally determinant in terms of association with poor outcome.
- Impossible to answer
- N/A (not within my expertise)

* 6. An episode of high CPP has a certain intensity (level of elevation), and a certain duration. Please indicate which of both you rate as most determinant in terms of association with poor outcome.

- For episodes of high CPP, the **intensity** (level of elevation) is more determinant than the duration in terms of association with poor outcome.
- For episodes of high CPP, the **duration** is more determinant than the intensity (height) in terms of association with poor outcome.
- For episodes of high CPP, the intensity (level of elevation) **AND** duration are equally determinant in terms of association with poor outcome.
- Impossible to answer
- N/A (not within my expertise)

* 7. Association with poor outcome has been reported for both low and high CPP. Please indicate which insult is most detrimental.

- Episodes of too low CPP are **more** detrimental than episodes of too high CPP.
- Episodes of too low CPP are **equally** detrimental than episodes of too high CPP.
- Episodes of too low CPP are **less** detrimental than episodes of too high CPP.
- Impossible to answer
- N/A (not within my expertise)

* 8. Randomized studies on protocols to target CPP above a fixed threshold failed to demonstrate benefit. Meanwhile, low CPP protocols in some centers were not associated with worse outcomes. Please choose which statement is most accurate.

- Because of potential dynamic CA impairment, absolute and universal CPP targets do not exist. The safe CPP zone can differ between individuals and can change within individuals.
- Absolute and universal CPP targets do exist regardless of intact/deficient CA. Failures to identify a universal safe CPP zone in clinical trials on severe TBI only reflect technical/methodological issues.
- Impossible to answer
- N/A (not within my expertise)

* 9. Please indicate which statement on the relation between CA status and CPP target zone is most accurate.

- The CPP target zone does not depend on CA status.
- The CPP target zone corresponds to the area between the lower and upper limit of CA. As CA can be dynamically impaired in severe TBI, CPP targets will depend on CA status.
- The CPP target zone depends on CA status as well as on other variables and is/can be narrower than the area between the lower and upper limit of CA.
- Impossible to answer
- N/A (not within my expertise)

As your reply to this question may depend on the clinical definition of CA you scored in question 1, you are free to comment.

* 10. It is safe to lower CPP all the way to the lower limit of CA, if this value could be reliably detected.

Very strongly disagree							Very strongly agree	N/A (not within my expertise)
○	○	○	○	○	○	○	○	○

* 11. It is safe to increase CPP all the way to the upper limit of CA, if this value could be reliably detected.

Very strongly disagree							Very strongly agree	N/A (not within my expertise)
○	○	○	○	○	○	○	○	○

Delphi consensus round 1 on the use of cerebrovascular autoregulation monitoring in adult severe TBI management

Role of autoregulatory (CA) status in management protocols in adult severe TBI.

Please note that all questions relate to the setting of adult severe TBI.

* 12. Please indicate which statement is most accurate on the potential role of CA status in CPP management protocols.

- ◯ CA status helps in choosing between primarily ICP targeted therapy (in case of deficient CA) or primarily CPP targeted therapy (in case of intact CA).
- ◯ CA status helps/could help in dynamically determining the safest CPP zone, applying so-called 'CPP opt' algorithms.
- ◯ The value of incorporating CA status in CPP management is insufficiently proven.
- ◯ N/A (not within my expertise)

* 13. When managing low CPP, lowering high ICP should have priority over increasing the mean arterial blood pressure (in other words, management of high ICP should have priority).

Very strongly disagree							Very strongly agree	N/A (not within my expertise)
◯	◯	◯	◯	◯	◯	◯	◯	◯

* 14. It is impossible to make specific recommendations for a hierarchical order or clinical priority in currently available multimodal monitoring signals.

Very strongly disagree							Very strongly agree	N/A (not within my expertise)
◯	◯	◯	◯	◯	◯	◯	◯	◯

Comment

[ ]

Delphi consensus round 1 on the use of cerebrovascular autoregulation monitoring in adult severe TBI management

Measurement/monitoring of autoregulatory (CA) status

Please note that all questions concern the setting of adult severe TBI.

Measurement of static CA

* 15. Please score the following measures of static CA according to their level of accuracy.

	Really very inaccurate							Really very accurate	N/A (not within my expertise)
ARI (the Autoregulation Index, defined as a dimensionless index ranging from 0 to 9, for which a response of transcranial Doppler based flow velocity (FV) to a hypothetical impulse change in arterial blood pressure (ABP)									
Transfer function analysis methods describing the relation between arterial blood pressure and transcranial Doppler based flow velocity									
Intracranial pressure response to arterial blood pressure manipulation									

* 16. Please score the following measures of static CA according to their level of reproducibility.

	Not at all reproducible							Really very reproducible	N/A (not within my expertise)
ARI (the Autoregulation Index, defined as a dimensionless index ranging from 0 to 9, for which a response of transcranial Doppler based flow velocity (FV) to a hypothetical impulse change in arterial blood pressure (ABP)									
Transfer function analysis methods describing the relation between arterial blood pressure and transcranial Doppler based flow velocity									
Intracranial pressure response to arterial blood pressure manipulation									

* 17. Please score the following measures of static CA according to their level of validation.

	Not at all validated							Very strongly validated	N/A (not within my expertise)
ARI (the Autoregulation Index, defined as a dimensionless index ranging from 0 to 9, for which a response of transcranial Doppler based flow velocity (FV) to a hypothetical impulse change in arterial blood pressure (ABP)	○	○	○	○	○	○	○	○	○
Transfer function analysis methods describing the relation between arterial blood pressure and transcranial Doppler based flow velocity	○	○	○	○	○	○	○	○	○
Intracranial pressure response to arterial blood pressure manipulation	○	○	○	○	○	○	○	○	○

Please comment on how you interpret 'validation'.

[ ]

Monitoring of dynamic CA

* 18. Please score the following measures of dynamic CA according to their level of accuracy.

	Really very inaccurate							Really very accurate	N/A (not within my expertise)
PRx (the Pressure Reactivity Index, calculated as the moving Pearson correlation coefficient between 30 consecutive 10 second averages of intracranial pressure and mean arterial blood pressure signals using waveform data capture)	○	○	○	○	○	○	○	○	○

	Really very inaccurate							Really very accurate	N/A (not within my expertise)
L-PRx (the 'long' Pressure Reactivity Index, calculated as the moving Pearson correlation coefficient between 20 consecutive values of intracranial pressure and mean arterial blood pressure using minute by minute signal capture)									
LAx (the Low-frequencey Autoregulation Index, the averaged moving Pearson correlation coefficient of minute by minute signals of intracranial pressure and mean arterial blood pressure calculated over time intervals varying between 3 and 120 minutes)									
Mx (the Mean Flow Index, calculated as the Pearson correlation coefficient between 40 consecutive 6 second averages of transcranial Doppler based mean flow velocity and cerebral perfusion pressure signals)									
ORx (the brain tissue oxygen pressure reactivity index, calculated as the Pearson correlation coefficient between signals of PbO2 and cerebral perfusion pressure every 30 seconds over intervals of 1, 6 or 12 hours)									

	Really very inaccurate							Really very accurate	N/A (not within my expertise)
Lx (the laser Doppler flow based autoregulation index, calculated as the Pearson correlation coefficient of 30 consecutive 10 second averages of Laser Doppler flow based cerebral blood flow and cerebral perfusion pressure)	◯	◯	◯	◯	◯	◯	◯	◯	◯
TOx (the Total Oxygen based CA index, calculated as the moving Pearson correlation coefficient of 30 consecutive 10 second averages of Near Infrared Spectroscopy based Total Oxygen Index and cerebral perfusion pressure)	◯	◯	◯	◯	◯	◯	◯	◯	◯
THx (the Total Hemoglobin based CA index, calculated as the moving Pearson correlation coefficient of 30 consecutive 10 second averages of Near Infrared Spectroscopy based Total Hemoglobin Index and cerebral perfusion pressure)	◯	◯	◯	◯	◯	◯	◯	◯	◯
The correlation of extracellular glutamate (as measured with microdialysis) and cerebral perfusion pressure	◯	◯	◯	◯	◯	◯	◯	◯	◯
CBFx (the thermal diffusion based autoregulation index, calculated as the Pearson correlation coefficient of 30 consecutive 10 second averages of thermal diffusion based cerebral blood flow and cerebral perfusion pressure)	◯	◯	◯	◯	◯	◯	◯	◯	◯

* 19. Please score the following measures of dynamic CA according to their level of reproducibility.

	Not at all reproducible							Really very reproducible	N/A (not within my expertise)
PRx									
L-PRx									
LAx									
Mx									
ORx									
Lx									
TOx									
THx									
Correlation extracell. glutamate and CPP									
CBFx									

* 20. Please score the following measures of dynamic CA according to their level of validation.

	Not at all validated							Very strongly validated	N/A (not within my expertise)
PRx									
L-PRx									
LAx									
Mx									
ORx									
Lx									
TOx									
THx									
Correlation extracell. glutamate and CPP									
CBFx									

Please comment on how you interpret 'validation'.

Generic autoregulation measurement/monitoring tool questions

* 21. Static CA measures are preferred over dynamic CA monitoring methods.

Very strongly disagree							Very strongly agree	N/A (not within my expertise)

* 22. Current tools to estimate CA status are insufficiently understood. The different indices produce different information.

Very strongly disagree							Very strongly agree	N/A (not within my expertise)
○	○	○	○	○	○	○	○	○

* 23. Implementing estimations of CA status in clinical practice is safe.

Very strongly disagree							Very strongly agree	N/A (not within my expertise)
○	○	○	○	○	○	○	○	○

* 24. The implementation of estimations of CA status in clinical decisions is still a theoretical concept.

Very strongly disagree							Very strongly agree	N/A (not within my expertise)
○	○	○	○	○	○	○	○	○

* 25. Information on CA status may be helpful, but is subordinate to ICP, CPP and PbO2 signals.

Very strongly disagree							Very strongly agree	N/A (not within my expertise)
○	○	○	○	○	○	○	○	○

Delphi consensus round 1 on the use of cerebrovascular autoregulation monitoring in adult severe TBI management

Association of autoregulatory (CA) status with outcome independent from perfusion variables

All questions concern the setting of adult severe TBI.

* 26. Impaired autoregulation worsens tolerability for high ICP (i.e. association with worse outcome occurs at lower ICP values).

Very strongly disagree							Very strongly agree	N/A (not within my expertise)
○	○	○	○	○	○	○	○	○

* 27. Impaired autoregulation worsens tolerability for low PbO2 (i.e. association with worse outcome occurs at higher PbO2 values).

Very strongly disagree							Very strongly agree	N/A (not within my expertise)
○	○	○	○	○	○	○	○	○

* 28. Impaired autoregulation worsens overall tolerability for secondary insults (i.e. unfavorably shifts the thresholds associated with worse outcome).

Very strongly disagree							Very strongly agree	N/A (not within my expertise)
○	○	○	○	○	○	○	○	○

* 29. Whether overall CA status is intact or deficient, has an independent association with outcome (regardless of actual CPP).

Very strongly disagree							Very strongly agree	N/A (not within my expertise)
○	○	○	○	○	○	○	○	○

Delphi consensus round 1 on the use of cerebrovascular autoregulation monitoring in adult severe TBI management

Research agenda on cerebrovascular autoregulation (CA)

All questions concern the setting of adult severe TBI.

* 30. The priority for research on CA is

- ○ low
- ○ moderate
- ○ high

Comments:

* 31. For a clinical measurement/monitoring method to represent CA requires

- ☐ validation against pial arteriolar diameter and flow velocity changes in an animal model.
- ☐ validation against mathematical models of CA.
- ☐ validation against outcome in prospective patient cohorts.
- ☐ Other (please specify)

* 32. Research focus should go to (please rate in terms of importance)

	Not at all important							Really very important	N/A (not within my expertise)
laboratory work for better understanding of CA physiology under healthy circumstances.	○	○	○	○	○	○	○	○	○

	Not at all important							Really very important	N/A (not within my expertise)
laboratory work for better understanding of CA impairment pathophysiology in brain injury.									
laboratory work for development of better CA measurement/monitoring methods.									
laboratory work for validation of existing clinical measurement/monitoring tools.									
mathematical modeling work for development of better CA measurement/monitoring methods.									
development of protocols to implement CA information in clinical situations.									
development of algorithms that translate CA information in dynamic CPP targets (CPPopt algorithms).									
prospective patient feasiblity studies to test protocols that incorporate CA information.									
prospective patient feasibility studies to test whether dynamic CPP targets from CPPopt algorithms can be achieved/maintained.									
prospective patient safety studies on the implementation of CA information in clinical situations.									
prospective patient safety studies on dynamic CPP targets from CPPopt algorithms.									
randomized controlled trials on ICP targeted versus CPP targeted therapy as determined by CA status.									

	Not at all important							Really very important	N/A (not within my expertise)
randomized controlled trials on dynamic CPP targets from CPPopt algorithms versus standard CPP management.	◯	◯	◯	◯	◯	◯	◯	◯	◯

33. Please freely enter any thoughts on CA research agenda.

Delphi consensus round 1 on the use of cerebrovascular autoregulation monitoring in adult severe TBI management

Level of expertise questions

* 34. I am a

- ◯ neuro-anesthetist
- ◯ neuro-intensivist
- ◯ neurosurgeon
- ◯ neurologist
- ◯ Other (please specify)

* 35. I have expertise in management of adult severe TBI for

- ◯ less than 5 years
- ◯ 5-10 years
- ◯ 10-20 years
- ◯ more than 20 years

* 36. my email address is (the email address will only be used for the next Delphi round):
